# Digital Content-Free Speech Analysis Tool to Measure Affective Distress in Mental Health: Evaluation Study

**DOI:** 10.2196/37061

**Published:** 2022-08-30

**Authors:** Peter Tonn, Lea Seule, Yoav Degani, Shani Herzinger, Amit Klein, Nina Schulze

**Affiliations:** 1 Neuropsychiatric Center of Hamburg Hamburg Germany; 2 VoiceSense Ltd Herzelia Israel

**Keywords:** mobile health, mHealth, depression, assessment, voice analysis, evaluation, speech, speech analysis, tool, distress, mental health, mood, diagnosis, measurement, questionnaire, mobile phone

## Abstract

**Background:**

Mood disorders and depression are pervasive and significant problems worldwide. These represent severe health and emotional impairments for individuals and a considerable economic and social burden. Therefore, fast and reliable diagnosis and appropriate treatment are of great importance. Verbal communication can clarify the speaker’s mental state—regardless of the content, via speech melody, intonation, and so on. In both everyday life and clinical conditions, a listener with appropriate previous knowledge or a trained specialist can grasp helpful knowledge about the speaker's psychological state. Using automated speech analysis for the assessment and tracking of patients with mental health issues opens up the possibility of remote, automatic, and ongoing evaluation when used with patients’ smartphones, as part of the current trends toward the increasing use of digital and mobile health tools.

**Objective:**

The primary aim of this study is to evaluate the measurements of the presence or absence of depressive mood in participants by comparing the analysis of noncontentual speech parameters with the results of the Patient Health Questionnaire-9.

**Methods:**

This proof-of-concept study included participants in different affective phases (with and without depression). The inclusion criteria included a neurological or psychiatric diagnosis made by a specialist and fluent use of the German language. The measuring instrument was the VoiceSense digital voice analysis tool, which enables the analysis of 200 specific speech parameters based on machine learning and the assessment of the findings using Patient Health Questionnaire-9.

**Results:**

A total of 292 psychiatric and voice assessments were performed with 163 participants (males: n=47, 28.8%) aged 15 to 82 years. Of the 163 participants, 87 (53.3%) were not depressed at the time of assessment, and 88 (53.9%) participants had clinically mild to moderate depressive phases. Of the 163 participants, 98 (32.5%) showed subsyndromal symptoms, and 19 (11.7%) participants were severely depressed. In the speech analysis, a clear differentiation between the individual depressive levels, as seen in the Patient Health Questionnaire-9, was also shown, especially the clear differentiation between nondepressed and depressed participants. The study showed a Pearson correlation of 0.41 between clinical assessment and noncontentual speech analysis (*P*<.001).

**Conclusions:**

The use of speech analysis shows a high level of accuracy, not only in terms of the general recognition of a clinically relevant depressive state in the participants. Instead, there is a high degree of agreement regarding the extent of depressive impairment with the assessment of experienced clinical practitioners. From our point of view, the application of the noncontentual analysis system in everyday clinical practice makes sense, especially with the idea of a quick and unproblematic assessment of the state of mind, which can even be carried out without personal contact.

**Trial Registration:**

ClinicalTrials.gov NCT03700008; https://clinicaltrials.gov/ct2/show/NCT03700008

## Introduction

### Background

Mental illnesses are highly important worldwide [1]. In particular, affective disorders such as depression or anxiety lead to considerable distress in individuals who are affected [2]. In addition, these disorders are an economic and social burden to society [3]. To recognize the need for care and the effects of ongoing treatment or treatment that may need to be adapted quickly, contact with mental health professionals (physicians, trained nursing staff, or psychologists) is essential [4]. Still, many of the patients were in primary care treatment without mental health specialization, only with short-time contact with a physician and did not reach complete remission [5]. In addition to the difficulty of getting an appointment with a professional, there is a problem of fluctuations in the symptoms [4], which makes it challenging to determine clinical symptoms. The calls to implement measurement-based treatment in psychiatry are increasing; however, clinician-rated assessments and patient-reported outcomes may be complex [6]. Therefore, the necessity for new assessment methods is undisputed, with which more frequent or even regular progress control is possible.

For several years, such assessments have been known as ecological momentary assessment (EMA) [7,8]. With the development of digital technologies, particularly smartphones, they have been extensively studied [9]. Conventional measurement methods are either digitized or mobilized. Diverse approaches have also been undertaken to develop and evaluate new measurement methods that were previously not feasible; for example, use of wearables measuring biological and behavioral indices such as temperature, movement, or heart rate [10]. Such procedures result in 2 advantages from the point of view of the practitioner: on the one hand, it is possible to collect findings from real life in a close-meshed manner [11]; on the other hand, the application of the technology enables the person concerned to do part of the recovery process himself and control what increases compliance [12]. The application of EMA allows a short assessment to be performed frequently and under conditions of everyday life [7].

The importance of language and voice in conveying feelings, emotions, and moods is undisputed, and impairments in speech production are well known in mood disorders [13-15]. The content of the spoken word (the “text”) [16] and nonverbal aspects (speaking style, flow of language, speech melody, etc) are both relevant and relatively independent [17-19]. Even without social interaction, inner speech is essential for the development of self-worth and self-efficacy, as well as for reflecting on experience and relational framing [20]. Human language as a form of expression, with human listening on the other side, has been the basis of professional therapeutic support for mental disorders for more than 100 years [21]. Recent developments in in linguistic and psychological research in relational frame theory have been acceptance-based treatments, such as acceptance and commitment therapy [22,23].

In the last few decades, advances in digital technology to diagnose or treat mood disorders have been enormous and have enabled a wide range of applications [24]. The worldwide spread of smartphones is so pronounced that an overwhelming proportion of people have access to this technology [25]. This also allows a better acquisition of voice recordings over a distance than was possible a few years ago, and the development of calculation power to push forward machine learning in different ways allows more intensive and detailed analysis of speech and voice [26,27]. By developing and applying unique algorithms, attempts can now be made to map the performance of active human listening and the associated assignment of emotions and moods [26]. This technique using machine learning should then be applied in the sense of an EMA in everyday life to record depressive moods and, ideally, observe the therapeutic effect and course [28].

It is well known that human speech is not simply the process of opening and closing the mouth but a more complex process involving more than 100 muscles, powered by many different brain regions, especially the auditory cortex, somatosensory cortex, and other brain parts and networks working in language perception and speech production [29]. There are many different acoustic features, such as pitch, speed, sound intensity, jitter, tremor, and vowel space, which are different in depressed patients compared with nondepressed persons. Research has been conducted to improve the knowledge of single-person aspects [30]. Thus, we have learned in the past that experienced clinicians or close friends can realize altered acoustic features in the case of depression, but it should be an essential perspective to implement accurate algorithms to analyze these parameters correctly without human supervision.

This study uses a noncontent linguistic analysis algorithm developed by VoiceSense, a company specializing in prosodic speech analysis, which links speech patterns to behavioral tendencies. Using machine learning technology to analyze data obtained from the speech or voice of patients with mental health complaints could be helpful to (1) complement the assessments of experienced psychiatrists or not-so-experienced physicians in primary care and (2) monitor the symptomatology of patients between consultations. This technology could optimize treatment procedures by learning the success rates of different individual treatments [31]. However, it is essential to train and check the machine learning algorithms and prove the algorithms repeatedly using samples with English or non-English native speakers, different mental states, or defined mental disorders in a longitudinal study [32].

This study provides a structured setting for the differentiated evaluation of psychopathological factors (effects, personality aspects, psychomotor factors, etc) using speech pattern analysis.

### Research Objectives

The primary objective of this study was to examine the depression severity state of patients with depression, as measured by the Patient Health Questionnaire-9 (PHQ-9), compared with their acoustic vocal patterns.

We hypothesized that the vocal analysis system could confirm depression severity classification performed by an experienced psychiatrist consultant.

## Methods

### Setup

#### Participants

Participants were recruited in an outpatient neuropsychiatric treatment center.

The inclusion criteria were neurological or psychiatric diagnosis made by a psychiatric consultant who was not part of the study team and fluent use of the German language. The diagnostics were based on the International Classification of Diseases, 10th Revision criteria because they are binding in the German public health care system and the psychiatric consultant’s experience.

Exclusion criteria included psychosis, dementia, speech or language disorders in neurological diseases, terminal or life-threatening illnesses, addiction history, a suicide attempt recently or in the last 12 months, or insufficient language skills.

Data collection took place between November 2018 and September 2019.

After removing participants who did not complete either the psychiatric assessment or audio collection, a total of 163 participants were included in the study, 47 (28.8%) of whom were men. We established a G-power calculation before starting the study and calculated the necessity of 152 participants to reach the required power.

#### Assessment Sessions

Each participant participated in at least one assessment session (psychiatric and voice). In contrast, a part of the participants (100/163, 61.3%) took part in 2 assessment sessions, and a part of the participants took part in 3 (29/163, 17.8%) assessment sessions. Because of our proof-of-concept study setting, we set a relatively open frame for the participants and assessments. The number of assessments carried out was determined by the follow-up appointments of the attending physicians; participants who had one or more outpatient appointments with the therapist within a short period were asked to participate in the next assessment more frequently.

The time intervals between the first, second, and third sessions for the same participant were at least one week, ranging up to 3 months, so that the psychiatric state and the voice patterns could be different in the session, even for the same patients. Therefore, each participant session was treated as a separate data point in the sample and included an independent psychiatric assessment and voice analysis.

#### Number of Assessments

Overall, there were 292 psychiatric assessments and 292 recorded audio sessions for the 163 participants. These 292 sessions (psychiatric and voice) consisted of the data set sample to compare the psychiatric state and vocal patterns.

#### Demographic Characteristics

The participants were aged between 15 to 82 years. The participants were categorized into 6 age groups to examine age-related differences. Table 1 shows the distribution of the participants and sessions across the demographic scales.

**Table 1:** Participants’ distribution across demographic scales (N=163) and sessions (n=292).

Demographic category		Participants, n (%)	Sessions, n (%)
**Gender**			
	Male	47 (28.8)	73 (25.0)
	Female	116 (71.2)	219 (75.0)
**Age group (years)**			
	15 to 20	8 (13.0)	17 (5.8)
	21 to 30	57 (35.0)	112 (38.4)
	31 to 40	46 (28.2)	81 (27.7)
	41 to 50	24 (14.7)	39 (13.4)
	51 to 60	21 (12.9)	32 (11.0)
	61 to 82	7 (4.3)	11 (3.9)
**Education level**			
	No completed education	38 (23.3)	70 (24.0)
	Apprenticeship	53 (32.5)	93 (31.8)
	Master craftsman certificate	7 (4.3)	16 (5.5)
	University degree	60 (36.8)	102 (34.9)
	Others	5 (3.1)	11 (3.9)
**Marital status**			
	Single	77 (47.2)	148 (50.7)
	Married or in a relationship	73 (44.8)	129 (44.2)
	Living apart or divorced	12 (7.4)	14 (4.8)
	Widowed	1 (0.6)	1 (0.3)
**Current psychological treatment**			
	No	60 (36.8)	124 (42.5)
	Yes	103 (63.2)	168 (57.5)

### Tools

#### Psychiatric and Behavioral Assessments

To differentiate and compare depressive states, we used PHQ-9 with 9 questions, developed and published in 1999 [33]. This assessment was initially created for the screening and measurement of severity. The PHQ-9 score ranges from 0 to 27, because each of the 9 items can be scored from 0 to 3. A score of 3 indicates severe symptoms, and 0 indicates an absence of symptoms. The authors of the PHQ-9 described a model that shows a high correlation between the 5 states of depression and between clinicians’ diagnosis and severity classification, whereas other authors replicated the quality of the tool [34]. Validation of the tool included 3890 patients. We used the PHQ-9 for both the diagnosis and the severity classification.

The PHQ-9 scale consists of 5 depression categories. Textbox 1 lists these 5 categories.

We also performed other measurements to compare vocational state with other mental problems or disorders, but the results will be published elsewhere.

**Patient Health Questionnaire-9 (PHQ-9) depression categories.**

**PHQ-9 scale and depression category**

- 0 to 4: absence of depressive disorder
- 5 to 10: subsyndromal symptoms
- 10 to 14: mild symptoms
- 15 to 19: moderate symptoms
- 20 to 27: severe symptoms

#### Audio Collection Software

The participants’ voices were recorded using the VoiceSense mobile audio collection app. VoiceSense mobile apps collect patients’ audio for health care monitoring. The app required a log-in based on the coded ID of the participant to prevent personal identification. After log-in, the participant was presented with general questions (eg, “Please say in a few sentences on your social life. How often do you meet with friends? Do you spend quality time with your family?”). There are 9 such general questions, and the appl displayed 1 out of the 9 questions every time. The participant was requested to press *record*, answer vocally to the presented question, and press *stop recording* when completed. If required, additional questions were raised until at least 2 minutes of the participant’s voice were collected. Once completed, the audio was uploaded to VoiceSense’s cloud server for analysis using a special algorithm. During audio collection, the participants were alone in one of our testing rooms.

#### Vocal Analysis

The VoiceSense behavioral vocal analysis software analyzed the recorded voices of the patients based on previous work to code and train a machine learning analyzer.

VoiceSense behavioral vocal analysis software applies acoustic analysis, focusing on the prosodic features of speech. The analysis was language independent. The analysis does not use speech recognition, so there is no understanding of what has been said; hence, it is entirely content-free. To help the participants speak freely and noncontentually, they were presented with a set of questions (Multimedia Appendix 1). They had the option of either answering these questions or expressing their ideas and opinions.

According to the rules of the European Union regulations or the Defense Advanced Research Projects Agency of the US-Military (DARPA), we describe the process of analytics a little bit more in the next sentences not as a *black box* but as a process with 3 steps:

1. VoiceSense vocal analysis first calculated over 200 raw voice parameters from the samples of each audio recording. These basic parameters consist of a wide range of acoustic feature segmentation, including lengths, ranges, slopes, frequencies, values, and shapes of pitch-extracted parameters, amplitude-extracted parameters, and silence-extracted parameters within the speech recording. Thousands of data points are calculated and averaged per recording to form a data set of over 200 parameters that reflects the individual’s speech patterns in the given recording.
2. The basic parameters were then calibrated and normalized to overcome possible biasing effects within the specific recording owing to amplitude differences, pitch differences, speech type differences (conversation or monologue), gender differences, and age differences. The calibration process was performed against large natural-speech vocal reference data sets with over 14,500 recordings collected by VoiceSense over the years, covering different speech types, genders, ages, and languages.
3. The calibrated and normalized parameters were then analyzed using machine learning techniques to select and weight the vocal parameters that best correlated with the searched phenomena. The process uses the standard
  repeated random subsampling cross-validation method
  , which randomly splits the data set into training and test subsamples and repeats the process for multiple iterations to obtain a stable and reliable predictive model equation. Therefore, this model provides a unified speech-based score with normalized continuous scores as well as categorized (1-10) scores, which are associated with the phenomena, in this case, with depression. The statistical fit of the model to the reference scale (in this case, depression) was evaluated using Pearson correlation, ANOVA, and positive and negative predictive values (confusion matrix).

### Procedures

#### Participant Recruitment

Participants were recruited from August 2018 to August 2019 at the Neuropsychiatric Center of Hamburg, one of Germany’s largest outpatient only treatment centers, focused on neurology and psychiatry.

All participants were patients treated at the Neuropsychiatric Center with a diagnosis of depression. During the recruitment period, the patients were informed of the possibility of their physicians’ participation in the study.

In all, 2 study supervisors (PT and NS) recruited the participants. The supervisors did not have any contact with the participants.

#### Baseline Assessment

After participants recruitment, a baseline assessment was taken, which include the items listed in Textbox 2.

The results of the Big-5 personality questionnaire and the attention deficit hyperactivity disorder–related diagnostics will be published later.

**List of items in baseline assessment.**

**Baseline assessment**

- Demographics (gender, age, psychological treatment, educational level, and marital status)
- Big-5 personality questionnaire for assessment of behavioral tendencies. The Big-5 questionnaires were administered only once, as unlike the psychiatric state, it was not expected to change from one session to the other.
- Attention deficit hyperactivity disorder (ADHD): some of the participants had already undergone ADHD assessment in the past. The ADHD diagnosis (yes or no) was added to the data set of the participants.

#### Psychiatric Assessment Session

In each assessment session, the participants completed the following psychiatric evaluation tools as listed in Textbox 3.

The participants completed the questionnaires. The assessments were performed after the session. The results of the General Anxiety Disorder-7 scale, Patient Health Questionnaire-15, and Symptom Checklist-90-R will be published in a separate publication.

**Psychiatric evaluation tools.**

**Evaluation tools**

- Patient Health Questionnaire-9 for depression
- General Anxiety Disorder-7 scale
- Patient Health Questionnaire-15 for somatic symptom severity, somatization, and somatoform disorders
- Symptom Checklist-90-R

#### Audio Collection Session

VoiceSense audio collection apps were installed on the tablets of the mental health professionals who participated in the research.

During each assessment session, the research assistant activated the VoiceSense app installed on the tablet. The app was logged in with the participant’s experiment ID in each session. However, the participant’s ID was only a serial number given to each participant by the research administrator. In this way, the recording could be associated with a specific participant but could not be associated with the actual identification of the participant. The conversion table between the actual participant’s ID and the ID of the experiment was kept secure by the research administrator.

After log-in, the app presented to the participant selected general questions (as described in the audio collections section). The participant was requested to answer the question, and the app recorded the participant’s voice while responding to the questions. The mental health professionals ensured that the recording was performed in a quiet environment (the session room), and only the participant’s voice was recorded. At least two minutes of audio was collected per participant in each session. If the recording length after the first question did not reach 2 minutes, the app presented another question or questions until at least two minutes of audio were collected.

#### Central Audio Analysis

Once the recording session was completed, the app sent the recorded audio to the VoiceSense cloud server. The VoiceSense cloud server is a highly secure server running under Microsoft Azure cloud facilities (in the United States) and is recognized by the German authorities as safe for keeping medical data.

VoiceSense software processes are certified by the ISO’s (International Organization for Standardization) highest information security standards, ISO 27001 and ISO 27799.

The audio data for each session were analyzed as described in the *Vocal Analysis* section, and over 200 raw speech parameters were generated for each recorded session.

These raw data parameters were calibrated and normalized to overcome possible biasing effects. The calibrated parameters of the 292 sessions were used as the input data set for statistical analysis.

#### Calculating the Vocal Depression Score

As described in the *Vocal Analysis* section of the *Methods* section, more than 200 raw voice parameters were calculated per audio recording. The parameters were calibrated and normalized by audio analysis and then passed as input data to the machine learning models for the vocal depression score calculation.

As described, a *repeated random subsampling cross-validation method*, using training and test subsamples, was used to select and weight the vocal parameters that best correlated with the searched phenomena (in this case, depression) to generate the articulated predictive model for depression.

Given the relatively small sample size of the study (N=292) compared with typical machine learning data sets, 5-fold cross-validation was chosen. The method randomly split the sample into a training subsample of 192 scores and a test subsample of 100 scores, and the process repeated itself for 10 iterations.

The method generated a predictive model (equation) consisting of a selected feature set of 13 weighted vocal parameters (out of over 200 input parameters). The outcome scores were normalized to generate a continuous vocal depression score (mean 5, SD 1). The score was also calculated on a categorical 1 to 10 scale (10=high depression).

### Ethics Approval

The study protocol was approved by the ethics committee of the Neuropsychiatric Center of Hamburg, Germany (No. 2017-002) under the Declaration of Helsinki. Written informed consent was obtained from all participants before inclusion in the study.

## Results

Overall, 163 participants completed both psychiatric assessments and audio collection, and 292 sessions had both psychiatric and vocal analysis scores.

### Psychiatric Scales Scores

The state of psychiatric severity could change between sessions and was, therefore, measured separately in every session. Hence, all 292 scores were used for psychiatric score comparisons, although there were only 163 participants in the study.

### Vocal Analysis Scores

The vocal analysis patterns could change between sessions and were, therefore, measured separately in every session. Hence, all 292 session scores were used for verbal and psychiatric score comparisons, although there were only 163 participants in the study.

### Correlation Between the Vocal Depression Score and the Psychiatric Depression Score (PHQ-9)

The overall Pearson correlations between the vocal depression score and the PHQ-9 depression score were highly significant, as presented in Textbox 4.

**Pearson correlations between the vocal depression score and Patient Health Questionnaire-9 for depression.**

**Pearson correlations**

- Overall sample (N=292): *r*=0.41; *P*<.001
- Training sample (N=192): *r*=0.46; *P*<.001
- Test sample (N-100): *r*=0.30; *P*=.001

### PHQ-9 (Depression)

#### Overview

Table 2 shows the PHQ-9 depression scores distributed by the 5 depression severity categories and the vocal analysis scores.

As can be seen, the average vocal depression score increases by the PHQ-9 depression severity category.

A single-factor ANOVA test was performed on the vocal depression scores and different PHQ-9 categories to determine whether the differentiation between the categories was significant. This result was highly significant (*F*_291_=14.5672; *P*<.001).

Specific 2-tailed *t* test comparisons were performed to determine the significance of the differences in vocal depression scores between each PHQ-9 depression severity category.

Table 3 shows the *t* test probability matrix of all the specific comparisons between the vocal depression scores of the different PHQ-9 severity categories. As can be seen, the vocal scores of all severity categories are significantly different from each other, except for the difference between categories 2 (subsyndromal symptoms) and 3 (mild symptoms) and between categories 4 (moderate symptoms) and 5 (severe symptoms).

**Table 2:** Depression (Patient Health Questionnaire-9 [PHQ-9]) scores by severity categories and vocal depression scores.

Overall sample (N=292)	Severity category				
	Absence of depressive disorder (1)	Subsyndromal symptoms (2)	Mild symptoms (3)	Moderate symptoms (4)	Severe symptoms (5)
Assessments, n	87	98	49	39	19
Vocal depression score, mean (SD)	4.521 (1.0929)	4.933 (0.841)	5.179 (0.829)	5.626 (0.913)	5.792 (0.963)

**Table 3:** *t* test probability matrix of vocal depression scores by Patient Health Questionnaire-9 (PHQ-9) severity category.

Depression (PHQ-9) score	Severity category				
	Absence of depressive disorder (1)	Subsyndromal symptoms (2)	Mild symptoms (3)	Moderate symptoms (4)	Severe symptoms (5)
Absence of depressive disorder (1)	1	0.0032^a^	9.89×10^−5b^	8.41×10^−9b^	1.6×10^−6b^
Subsyndromal symptoms (2)	—^c^	1	0.1155	7.26×10^−5b^	0.0004^b^
Mild symptoms (3)	—	—	1	0.0145^d^	0.0149^d^
Moderate symptoms (4)	—	—	—	1	0.5238
Severe symptoms (5)	—	—	—	—	1

^a^*P*<.01.

^b^*P*<.001.

^c^Not applicable.

^d^*P*<.05.

#### Comparison of PHQ-9 (Depression) Score and Vocal Depression Score Across Demographic Scales.

Table 4 shows the average PHQ-9 depression and the average vocal depression scores across the demographic scales.

Single-factor ANOVA tests were performed for each demographic scale to determine whether the differences between the scale categories were significant for both the PHQ-9 depression scores and vocal depression scores.

**Table 4:** Patient Health Questionnaire-9 (PHQ-9) and vocal depression scores by demographic scales (N=163 Participants, n=292 sessions).

Demographic category					Participants, n (%)		Sessions, n (%)		Depression (PHQ-9) score, mean (SD)		Vocal depression score, mean (SD)
**Gender**											
			Male	47 (28.8)		73 (25.0)		2.47 (1.281)		5.18 (1.050)	
			Female	116 (71.2)		219 (75.0)		2.29 (1.194)		4.94 (0.978)	
**Age group (years)**											
			15 to 20	8 (4.9)		17 (5.8)		1.765 (0.970)		4.580 (0.711)	
			21 to 30	57 (35.0)		112 (38.2)		2.027 (1.102)		4.881 (1.053)	
			31 to 40	46 (28.2)		81 (27.7)		2.432 (1.060)		4.991 (0.964)	
			41 to 50	24 (14.7)		39 (13.4)		2.949 (1.503)		5.245 (0.908)	
			51 to 60	21 (12.9)		32 (11.0)		2.844 (1.370)		5.547 (0.917)	
			61 to 82	7 (4.3)		11 (3.8)		1.909 (0.701)		4.471 (0.925)	
**Educational level**											
		No completed education			38 (23.3)		70 (24.0)		2.086 (1.176)		5.054 (0.951)
		Apprenticeship			53 (32.5)		93 (31.8)		2.753 (1.282)		5.231 (0.943)
		Master craftsman certificate			7 (4.3)		16 (5.5)		1.563 (0.629)		4.419 (0.904)
		University degree			60 (36.8)		102 (34.9)		2.216 (1.087)		4.890 (1.034)
		Others			5 (3.1)		11 (3.9)		2.545 (1.635)		4.567 (1.129)
**Marital status**											
	Single			77 (47.2)		148 (50.7)		2.446 (1.203)		5.024 (1.032)	
	Married or in a relationship			73 (44.8)		129 (44.2)		2.225 (1.270)		4.967 (0.972)	
	Living apart or divorced			12 (7.4)		14 (4.8)		2.071 (0.730)		4.977 (0.962)	
	Widowed			1 (0.6)		1 (0.3)		3.000 (-)		6.039 (-)	
**Current psychological treatment**											
	No			60 (36.8)		124 (42.5)		1.653 (0.817)		4.652 (0.865)	
	Yes			103 (63.2)		168 (57.5)		2.833 (1.222)		5.257 (1.017)	

### Gender Differences

No significant differences were found between men and women for either the PHQ-9 scores (ANOVA test: *F*_291_=1.173611; *P*=.28) or vocal depression scores (ANOVA test: *F*_291_=3.175943; *P*=.08).

### Age Group Differences

Significant differences in depression scores were found between age groups for both the PHQ-9 (ANOVA test: *F*_291_=6.162602798; *P*<.001) and vocal depression (*F*_291_=4.125516; *P*<.001) Depression scores were generally higher in older age groups for both PHQ-9 and vocal depression, except for the oldest age group.

**Figure 1:**
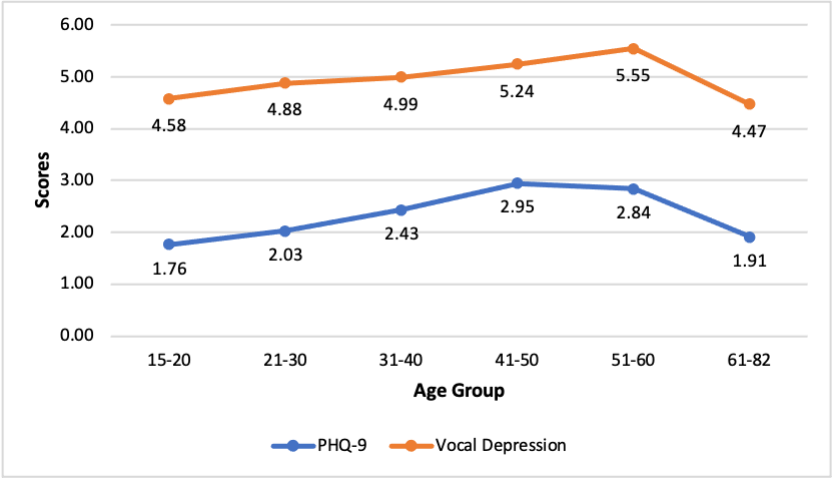
Patient Health Questionnaire-9 (PHQ-9) and vocal depression scores by age group.

As seen in Figure 1, the average score changes between age groups were very similar for PHQ-9 and vocal depression scores (the average scores are different owing to the different scales).

### Educational Level Differences

Significant differences in depression scores were found between educational level groups for both PHQ-9 (ANOVA test: *F*_291_=5.769297; *P*<.001) and vocal depression (*F*_291_=3.593119; *P*=.007).

**Figure 2:**
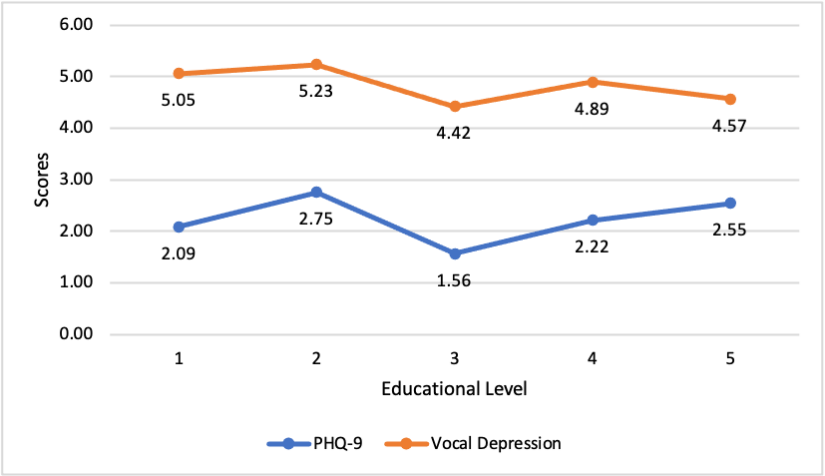
Patient Health Questionnaire-9 (PHQ-9) and vocal depression scores by educational level.

As seen in Figure 2, the average score changes for education levels were very similar for the PHQ-9 and vocal depression scores (the average scores are different owing to the different scales).

### Marital Status Differences

No significant differences were found in depression scores between the marital status groups, neither for the PHQ-9 (ANOVA test: *F*_291_=1.081893; *P*=.52) nor for vocal depression scores (ANOVA test: *F*_291_=0.434517; *P*=.73).

### Psychological Treatment Differences

#### Overview

Significant differences in depression scores were found between psychological treatment status for both the PHQ-9 (ANOVA test: *F*_291_=86.937647; *P*<.001) and vocal depression (ANOVA test: *F*_291_=28.66407; *P*<.001).

**Figure 3:**
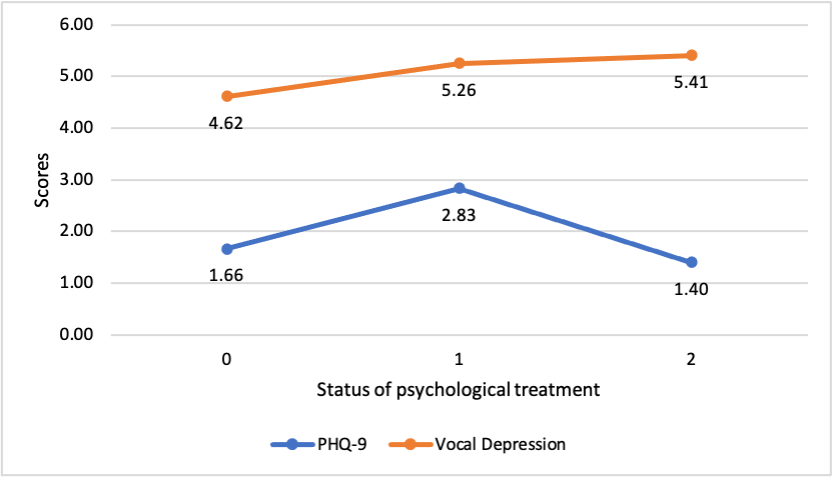
Patient Health Questionnaire-9 (PHQ-9) and vocal depression scores by psychological treatment status.

As seen in Figure 3, the average score changes for current psychological treatment status *no* in every sessions (0) and *yes* in every session (1) are similar for the PHQ-9 and the vocal depression scores (the average scores themselves are different due to the different scale), the average score changes are not clear similar in *no* in one session (2), but this group is too small with N=5 to allow a clear explanation.

#### Statistical Fit (Predictive Power) of the Vocal Depression Scores to the PHQ-9 Depression Scores

As shown by the aforementioned results, strong and significant relationships were found between the vocal depression scores and PHQ-9 depression scores.

However, what is the practical accuracy that can be expected from using vocal analysis for tracking and screening for depression? In statistical terms, this question relates to the predictive power of the vocal model or its statistical fit to PHQ-9 depression reference scores.

A common way to evaluate a model’s strength is by labeling the model’s scores as positive and negative predictive values and performing a confusion matrix analysis.

The PHQ-9 scores were labeled as follows: scores 1, 2, and 3 were labeled as *Low Depression* and scores 4 and 5 were labeled as *High Depression*. A total of 63.4% (185/292) of participants were labeled as *Low Depression*, and 36.6% (107/292) of participants were labeled as *High Depression*.

The vocal depression scores were labeled as follows: scores 1 to 6 (within the 1-10 scale) were labeled as *Low depression risk* and scores 7 to 10 were labeled as *High depression risk*. A total of 59.9% (175/292) of participants were labeled *Low depression risk*, and 40.1% (117/292) of participants were labeled as *High depression risk*.

Table 5 shows the confusion matrix of the number of participants classified according to these 4 labels for the entire sample and training and test subgroups.

Of the 107 participants that were labeled as *High Depression* according to the PHQ-9, 68 (63.6%) were classified as *High depression risk* by the vocal depression analysis (true positive) and 39 (36.4%) were classified as *Low depression risk* (false negative).

Out of the 185 participants that were labeled *Low Depression* according to the PHQ-9, 136 (73.5%) were classified as *Low depression risk* by the vocal depression analysis (true negative) and 49 (26.5%) were classified as *High depression risk* by the vocal depression analysis (false positive).

Overall, out of the 292 participants, 204 (136+68) were classified as consistent with the PHQ-9 depression (accuracy=69.9%).

Table 6 provides the confusion matrix attributes for the entire sample and training and test subgroups.

As explained in the *Methods* section, the vocal depression model was developed using a training subsample (on which the model was trained) and a test subsample (on which the model was tested).

Hence, the model's predictive power is best evaluated using the confusion matrix results for the test subsample. It is also interesting to observe the differences between the training and test subsamples to assess the expected stability of the model.

As can be seen, the confusion matrix results for the test subsample were similar to the results of the training subsample. The overall accuracy of the model was 70.8% in the training subsample and 68% in the test subsample. This means that the vocal depression model is relatively stable and is expected to provide an overall accuracy of close to 70% when used for depression screening and tracking.

**Table 5:** Patient Health Questionnaire-9 (PHQ-9) depression and vocal depression labeled matrixa.

Confusion matrix (vocal depression)	PHQ-9 depression (N=292)				Training subgroup (N=192)				Test subgroup (N=100)				
	Low	High	Total	Low		High	Total	Low		High	Total		
Low risk	136	39	175	89.2		24.7	113.9	46.8		14.3	61.1		
High risk	49	68	117	31.3		46.8	78.1	17.7		21.2	38.9		
Total	185	107	292	120.5		71.5	192	64.5		35.5	100		

^a^The values are participants and virtual parts of participants predicted and classified from the algorithm.

**Table 6:** Confusion matrix attributes.

	Entire sample (%)	Training subgroup (%)	Test subgroup (%)
Accuracy: correct classifications	69.9	70.8	68.0
Sensitivity (recall): true positive rate	63.6	65.5	59.7
Specificity: true negative rate	73.5	74.0	72.6
Precision: positive predictive value	58.1	59.9	54.5
False positive rate	26.5	26.0	27.4
False negative rate	36.4	34.5	40.3

## Discussion

### Principal Findings

In this study, consisting of 163 participants affected by different degrees of depressive states, it was shown that there is a high correlation between the depression severity classification as measured by the PHQ-9 self-filled questionnaires and between the depressive vocal scores as measured by the VoiceSense analysis system.

Compared with other studies that used biomarkers or wearables for detecting depressive states [35], we only used noncontent speech analysis. No participant had to buy or wear an additional digital tool or some hardware; no participant had to be aware of some unexpected action of an unusual wearable device. To start the analysis, it was only necessary to perform one short call with the VoiceSense system. The implementation of the VoiceSense speech analysis was unproblematic; the connection via Wireless Local Area Network showed adequate sending and receiving performance for the tablets used for recording. The sound quality was good and enabled a fair vocal analysis evaluation.

Each depressive vocal score was calculated based on only one voice recording in the evaluation. It is expected that the accuracy will increase with multiple recordings. Corresponding calculations will be performed for participants who have made several sound recordings.

As the basis for speech analysis, we used a machine learning algorithm that had already been applied to various personality aspects and affective states in English-speaking participants. Here, we tested whether and to what extent it is possible to let this system *learn further* to change the language and to what time the recognition of affective states can succeed in comparison with clinical tests. We achieved a high level of agreement with at least 384 training minutes after running the algorithm 10 times. The results of the confusion matrix showed that fairly good accuracy was achievable. The accuracy of the test sample was maintained at 68%. Specificity and sensitivity also correspond to high values of 59% and 72%, respectively. Even in everyday clinical practice, no better agreements are achieved by different therapists in cursory examinations.

We were able to achieve stable results with the voice analysis system, which largely corresponded to the results of testing in the PHQ-9. The effects were long-lasting; after training on the first 192 data sets, the following data sets could be detected with high accuracy. No restrictions due to the course of time or abnormalities in the intervals between individual tests were recognizable. Thus, the use of the speech analysis system alone could accurately discriminate individuals with a high risk of depressive phases or could monitor the course of treatment and recovery of patients without direct contact with an experienced psychiatrist.

### Comparison With Previous Research

We compared our study to other studies that investigated the approach to analyzing speech in patients affected by depression. Some actual research studies noncontent aspects of speech compared with low or severe depressive symptoms [36]. The results confirm a relationship between acoustic parameters of speech not only in the case of severe depressive disorder but also in nonsevere cases. This study used humans' interpretation of audio recordings; we used digital techniques for a similar task. However, some research has been conducted to develop machine learning settings to identify if someone is depressed. In the various conferences of Audio Visual Emotion Challenge and Workshop in recent years, based on different data sets of depressed people, some researchers have shown that automatized techniques to identify this subgroup could reach a good recognition level [37,38].

In a group of older adults with depression, voice pattern and speech activity could be shown in an automated deep learning–based analysis as essential parameters to detect late-life depression [39].

The results that we were able to find here correspond to the descriptions of the specified studies. The main strength of our study is that data from an average outpatient group were available from an outpatient care center. None of the test participants were first made aware of the study or the problem of depressive disorders and how external stimuli could measure them. Therefore, the results can be regarded as reliable.

Nevertheless, the experimental results of the researchers cited are confirmed. With a training effort of only 192 candidates, we achieved almost congruent results for the remaining 100 measurements in the test run. Therefore, the use of the technical approach described here for easier and faster diagnosis of depressive disorders can be considered helpful in clinical practice, especially in detecting depressive disorders at an early stage or monitoring close-meshed treatment.

An interesting study showed that a measurement-driven therapy approach for patients with depression (as part of a telemedicine platform) can achieve far better treatment results than the previous approach, with occasional live contact with the treating physician. Suppose you transfer the core idea here—the regular and low-threshold recording of treatment effects and progressions—to implementation in everyday therapy, independent of a telemedicine treatment platform. There should also be exciting improvements in the treatment quality if changes in findings can be applied promptly [40].

Such an automated analysis with a shallow threshold to commit will make it possible to provide a more intensive and individual, person-centered treatment if the measurement results show a highly negative mood or other problematic stages of depressive disorder. It can assist in regulating treatment and medication dosage more accurately according to the patient’s state at the time of vocal assessment.

### Conclusions

The PHQ-9 is a well-established and appropriate tool for screening and assessing depressive disorders and depressive phases. However, the PHQ-9, similar to other assessment tools, is problematic for some patients and time consuming for both physicians and patients.

Using a tool with machine learning algorithms, such as VoiceSense, to capture the affective phase of a patient can work not only much faster but also over distance because the patient and physician do not need to see and sit together. VoiceSense could be installed on the patient's smartphone so that only the scores are transmitted to the physician’s database. Treatment flexibility is possible, and distance diagnostics can be used.

The importance of such an assessment is not a considerable question—both to improve individual therapy planning and to distinguish threatening phases of depression of an individual from harmless ones with significantly reduced effort. The value of such a remote monitoring approach has recently been emphasized during the COVID-19 pandemic.

In a further step, possible changes in the course of therapy must be recorded more precisely. The extent to which the result of the speech analysis changes if the condition of the test participant changes, and of course, it must ultimately be confirmed whether the good correlation is also confirmed if the recording of the test participant’s condition is not only proved by a self-rating similar to the PHQ-9 but also through a suitable third-party rating. That is now reserved for future studies; we will conduct this in collaboration with one of the largest German public health insurance companies.

## Appendix

Multimedia Appendix 1 List of the general questions presented in the VoiceSense mobile audio collection app.

## Abbreviations

EMA: ecological momentary assessment
ISO: International Organization for Standardization
PHQ-9: Patient Health Questionnaire-9
