# Supplementary material for: Digital Content-Free Speech Analysis Tool to Measure Affective Distress in Mental Health: Evaluation Study
Source: JMIR Form Res. 2022 Aug 30;6(8):e37061. doi: 10.2196/37061 (PMC9472064; doi:10.2196/37061)
Supplement: Multimedia Appendix 1 [file formative_v6i8e37061_app1.docx]

**Appendix 1: List of the general questions presented in the Voicesense mobile audio collection application**

| 1. Please say in a few sentences how was your day yesterday. What did you do, how did you feel? |
| --- |
| 2. Please speak briefly about any topic that is on your mind today. Work, friends, family or anything else. |
| 3. Please say a few sentences about your hobbies. Do you like Sports? TV? Reading? Other? What do you like about it? |
| 4. Please speak briefly about the recent week. What did you do? Did anything special happen? |
| 5. Please say a few sentences about your social life. How often do you meet with friends? Do you spend quality time with your family? |
| 6. Please say in a few sentences what are your plans or expectations for tomorrow or the near future. Are there any preparations? |
| 7. Please describe in a few sentences your daily routine. Work, leisure, meals, times. How do you feel about your daily routine? |
| 8. Please say a few sentences about the kind of music that you like or the type of movies that you prefer. What do you like about it? |
| 9. How do you feel about life in general? Are you satisfied? Would you change anything? |
